# *Zéro allergie* research clinic: a clinical and research initiative in oral immunotherapy for managing IgE-mediated food allergy

**DOI:** 10.1186/s13223-024-00921-8

**Published:** 2024-11-02

**Authors:** Bénédicte L. Tremblay, Philippe Bégin, Frédérique Gagnon-Brassard, Anne-Marie Boucher-Lafleur, Marie-Ève Lavoie, Anne-Marie Madore, Sarah Lavoie, Cloé Rochefort-Beaudoin, Claudia Nuncio-Naud, Charles Morin, Guy Parizeault, Catherine Laprise

**Affiliations:** 1 Département des sciences fondamentales, Université du Québec à Chicoutimi, 555 Boulevard de l’Université, Saguenay, QC G7H 2B1 Canada; 2 Centre intersectoriel en santé durable, Université du Québec à Chicoutimi, Saguenay, QC Canada; 3 Division d’immunologie clinique, de rhumatologie et d’allergie, Département de pédiatrie, Centre hospitalier universitaire Sainte-Justine, Montréal, Canada; 4 Clinique recherche Zéro allergie UQAC - Centre intégré universitaire de santé et de services sociaux du Saguenay–Lac-Saint-Jean, Saguenay, QC Canada

**Keywords:** Asthma Desensitization, Epigenetics, Food allergy, Genetics, Gene expression, Metabolomics, Microbiota, Oral immunotherapy Public Health

## Abstract

**Background and methods:**

The *Zéro allergie* research clinic (Saguenay, Canada) is a clinical and research initiative in oral immunotherapy (OIT) for managing IgE-mediated food allergy (FA). A total of 183 children with FA and 27 non-allergic siblings were recruited to date in the *Zéro allergie* cohort (ZAC) to better understand biological mechanisms underlying FA and OIT prognosis. The primary aims are to (a) better understand the genetic, epigenetic, transcriptomic, metabolomic, and microbial diversity associated with FA; (b) establish the multi-omics and microbial diversity profiles of children following OIT to identify predictive prognosis biomarkers, (c) make OIT more accessible to the population of the *Saguenay–Lac-Saint-Jean* region, and (d) build a biobank of data and biological material.

**Results:**

The ZAC constitutes a unique and rich biobank of biological samples (blood, buccal swabs, microbiota samples [intestinal, buccal, nasal, and cutaneous]) combined with clinical data and more than 75 phenotypic characteristics.

**Conclusions:**

This represents an innovative interdisciplinary initiative by researchers, allergists, and paediatricians to make FA care accessible to a greater number of children with IgE-mediated FA. Ultimately, it will contribute to provide more accessible treatment options with greater chances of success through a better understanding of the biological nature of FA and OIT.

**Supplementary Information:**

The online version contains supplementary material available at 10.1186/s13223-024-00921-8.

## Introduction

Food allergy (FA) is a major public health concern with an increasing prevalence now reaching 5–10% of the population in the Western countries [1–3]. Moreover, as many as 40% of children with FA are allergic to more than one food [4]. Physical manifestations range from mild symptoms to life-threatening anaphylaxis. The current standard of practice for FA management relies on strict avoidance of the allergen(s) and treatment of allergic reactions with epinephrine autoinjectors [5]. Constant vigilance for accidental exposure is a source of anxiety that greatly impacts the quality of life and increases the risk of social isolation, psychological burden, and nutritional deficiencies [5–7].

Oral immunotherapy (OIT) is another management option that helps mitigate the risk of allergic reactions and potentially reduce food-related anxiety and improve patients’ sense of control [8]. OIT involves daily ingestion of a small initial dose of the allergenic food protein in which the dose is gradually increased over time following a treatment protocol. The goal is to achieve desensitization to a daily dose of the allergen, while providing protection against accidental trace exposures or contamination [8]. Clinical desensitization is the ability to increase the patient’s threshold dose, which is the minimum amount of allergen necessary to cause an allergic reaction. This clinical response depends on appropriate allergen exposure, which means that exposure discontinuation may bring the threshold dose back to its initial level [9]. Furthermore, a variable proportion of patients will also tolerate any serving of the allergen (complete desensitization) while some will achieve sustained unresponsiveness (SU), which is defined as the ability to safely consume any amount of foods containing the allergen even after a prolonged period of avoidance [9]. Although nearly 75% of individuals will achieve complete desensitization, only a third of those will achieve SU [10]. Notably, achieving SU appears to be influenced by the duration of OIT. A double-blind, placebo-controlled trial egg-OIT produced SU to 5 g protein for 28% of patients in egg-OIT group after two years [11], and for 50% of patients after four years [12]. However, all participants who completed the SU oral food challenge (75% of cohort) were still able to tolerate a higher egg dose on SU testing compared to their baseline. This suggests that sustained protection, although partial, persists in most if not all patients.

According to an International Delphi consensus, odds of OIT outcomes are variable, poorly predictable, and may depend on the specific allergen [13]. The development of FA can be influenced by several factors including genetic, epigenetic, environmental, metabolomic, and microbiota-related factors, all of which may contribute to a better understanding of FA and OIT prognosis [14, 15]. Studies have identified several genetic [16] and epigenetic [17] associations with FA, but very few have investigated epigenetic changes following OIT [18, 19]. The impact of OIT on gut microbiota was evaluated in peanut-allergic adults [20] and in cow’s milk-allergic children [21]. Only one study reported the impact of genetic variations on the prognosis of OIT [22]. Finally, only one study evaluated plasma metabolomic profiles in response to OIT [23]. They reported that bile acids and histidine were higher in individuals with SU compared to individuals with clinical desensitization [23].

Accessibility is one OIT’s primary challenges as it is generally limited both in urban regions, where there are not enough allergists to meet demand, and in rural regions, where there are few or no allergists [8]. According to the 2020 Canadian Society of Allergy and Clinical Immunology guidelines [8], one way to increase OIT accessibility is by offering training and support to paediatricians and family doctors, enabling them to provide OIT services under the supervision of an allergist. This approach has been implemented in the *Zéro allergie* research clinic in Saguenay, confirming its feasibility.

The *Zéro allergie* research clinic, established in 2020 in Saguenay (Canada), is a major intersectoral infrastructure of research and clinic consisting of physicians, research nurse, registered dietitian, researchers, research professionals, numerous research trainees, and patient partners (i. e. a patient, family member, or caregiver who collaborates with healthcare professionals to improve the quality of care). The overarching goal of the *Zéro allergie* cohort (ZAC), which includes recruited children from the *Zéro allergie* research clinic, is to better understand biological mechanisms underlying FA and successful OIT. The ZAC aims to (a) better understand the genetic, epigenetic, transcriptomic, metabolomic, and microbial diversity associated with FA; (b) establish the multi-omics and microbial diversity profiles of children following OIT to identify predictive biomarkers of success, (c) make OIT more accessible to the population of the *Saguenay–Lac-Saint-Jean* (SLSJ) region, and (d) build a biobank of data and biological material available for researchers. The ZAC will contribute to better understand the biological nature of FA as well as support the development of new therapeutic approaches.

## Methods

### Cohort

The *Zéro allergie* research clinic is affiliated to the *Centre intégré universitaire de santé et de services sociaux* (CIUSSS) *du *SLSJ and the *Université du Québec à Chicoutimi* (UQAC). The clinic is a clinical and research OIT initiative established by Professor Catherine Laprise (UQAC) and paediatricians (CIUSSS of SLSJ) with the guidance of Dr Philippe Bégin, allergist at the *Centre hospitalier universitaire Sainte-Justine*.

We recruited children from the SLSJ region in Quebec (Canada) suffering from IgE-mediated FA, who had already been referred for OIT by their paediatrician and met eligibility criteria. To be eligible, children had to undergo the OIT protocol and have three FA or less. Children with four or more FA at recruitment were excluded from the *Zéro allergie* research clinic and the ZAC. These children were treated at the hospital’s OIT clinic (CIUSSS of SLSJ) for safety and practical reasons. Diagnoses of FA were confirmed by a paediatrician based on clinical symptoms and skin prick test (wheal diameter ≥ 3 mm of the negative control, measured after 10 min) [24]. Follow-ups at the *Zéro allergie* research clinic were carried out by a paediatrician and a clinical nurse. Children undergoing the OIT protocol at the clinic can also consent to be part of the ZAC. A total of 217 allergic children and 27 siblings were recruited in the ZAC as of June 2024. At recruitment, a clinical standardized health and environmental questionnaire was administered to children and their parents. This questionnaire, derived from the statement of the American Thoracic Society [25], evaluates asthma, allergies, medication, health habits, family history, environmental exposures, and comorbidities. Blood, buccal (swab when blood sampling was unsuccessful) and stool samples were collected before and after OIT. Serum total and specific IgE levels, skin prick test results, blood cell counts, OIT dosing schedule, progress notes as well as antibiotic prescriptions were available. More than 75 phenotypic characteristics were listed for participants. Informed consent was obtained from all participants’ legal guardians at recruitment. The experimental protocol was approved by the ethics committees of the CIUSSS of SLSJ (project # 2022-015) and UQAC (UQAC: 2023 − 820).

### Oral immunotherapy protocols

*Zéro allergie* research clinic offers OIT services to the paediatric population. Preschool age represents a window of opportunity for well-tolerated and effective OIT [26]. In the clinic, sublingual immunotherapy (SLIT) was initiated to treat children waiting for OIT, in order not to miss the window of opportunity for inducing clinical remission. The waiting list is now considerably reduced and almost all children start OIT immediately after referral in the SLSJ region. The success of the OIT is defined by a clinical desensitization to the food allergen allowing the consumption of the maintenance dose of the allergen without any allergic reaction, while on therapy.

Two protocols of dosing schedule (standard dosing and double dosing protocols) have been optimized in collaboration with an allergist and according with the 2020 Canadian Society of Allergy and Clinical Immunology guidelines [8]. The standard dosing protocol was adapted by an allergist from the one published by Burks et al. [11]. It begins with an initial dosage between 0.625 and 2.5 mg of total allergen protein (5 mg if children are taking omalizumab) and doses are increased every two weeks to reach a dose of 400 mg of allergenic protein by allergen after about eight months of treatment. Children of all ages, with multiple FA and variable specific IgE/total IgE ratio followed this protocol. Another protocol using double dosing has been optimized and tested by the allergist of the clinic [8]. This protocol also begins with an initial dose between 0.625 and 2.5 mg of total allergen protein. Dose increases are doubled but occurred every two months to reach 400 mg of allergenic protein by allergen after about 18 months. Children aged 5 and under with a single FA followed this modified protocol. Children over 5 years old with a specific IgE/total IgE ratio < 20% were also eligible for this protocol. The main advantage of this protocol is that it requires fewer clinic visits and that it is possible to follow more children at a time. The potential disadvantage of this protocol is that children may be less tolerant of the doubled dose increases. In practice at the clinic, there were no differences in reactions to dose increases between the two protocols. The initial dosage depends on several factors including the severity of the FA. Most importantly, dosing protocols should be adapted to children response and personal objectives [8]. Protocols are both followed by a maintenance phase of 18 months where children consume 400 mg of allergic protein by allergen daily.

### Biological samples

Samples of blood, buccal DNA, and intestinal microbiota were taken from participants during their OIT. Data from clinical tests and observations related to their OIT were available through the *Zéro allergie* research clinic and the CIUSSS of SLSJ medical records. Blood samples were collected before and after OIT to extract DNA for genetic and epigenetic data as well as RNA for transcriptomic data. Plasma was separated from blood for metabolomic data. After processing, the samples were stored at -80 °C for subsequent analyses. Several sterile HydroFlock were used to collect buccal, cutaneous, and nasal samples. A total of four buccal swabs were used to collect DNA and three other buccal swabs were used for buccal microbiota. The swab was rubbed on the inside of the child’s cheek for 30 s and then placed in a microtube containing 1 ml of phosphate-buffered saline. For cutaneous microbiota, a swab was rubbed 50 times in the antecubital fossa. For nasal microbiota, a swab was rubbed in each nostril. Samples were stored at -80 °C until the DNA extraction. Stool samples were collected before and after OIT on sterile toilet paper folded in aluminum foil provided in a kit prepared by the research team and according to a collection protocol also provided to parents. Samples were frozen at home until their next visit to the clinic and then stored at -80 °C.

### Forthcoming multi-omics data acquisition

#### Genetic data

DNA will be extracted from buccal swabs using the Qiagen QIAamp DNA Mini extraction kit following the company’s instructions (Qiagen, Toronto, ON, Canada). DNA will also be extracted from blood using Qiagen DNeasy Blood and Tissue extraction kits. Genotypes will be obtained from whole-genome sequencing.

#### DNA methylation data

Genomic DNA will be extracted from blood using Qiagen DNAeasy Blood and Tissue extraction kits. DNA methylation analysis will be performed using whole-genome bisulfite sequencing.

#### Gene expression data

Blood samples were also collected into PAXgene™. Total RNA will be extracted from whole blood using PAXgene Blood RNA Kit (Qiagen, Toronto, ON, Canada). The expression levels in copy number of each gene will be obtained with RNA sequencing.

#### Metabolomic data

Untargeted metabolomic approach will be used to measure plasma metabolites using reverse phase chromatography and hydrophilic interaction liquid chromatography.

#### Microbiota data

DNA will be extracted from buccal, cutaneous, nasal, and stools samples using the DNeasy Powersoil Pro Kit (Qiagen, Toronto, ON, Canada). Sequencing of the microbiome will be carried out using shotgun metagenomic sequencing.

### Statistical analysis

R software v4.4.0 (R Foundation for Statistical Computing; http://www.r-project.org) [27] was used to test changes in skin prick tests before and after the OIT using the R function “glm” in “stats” package. Model was adjusted for sex, age, and type of allergen. Figures were created with BioRender.com.

## Results

### Recruitment

A total of 238 children with IgE-mediated FA followed OIT between 2020 and 2024 at the *Zéro allergie* research clinic in Saguenay, Canada. Of these, 217 agreed to participate in the ZAC, representing a participation rate of 91.2%. Among them, 14 agreed to the research but had not yet started OIT, nine had resolution of their FA before reaching the maintenance dose, and 11 abandoned OIT for various reasons (side effects, anxiety, compliance and logistic issues). Thus, 183 children participated in the ZAC. A total of 27 non-allergic siblings were also recruited. Recruitment is still ongoing.

### Oral immunotherapy (OIT) protocol and sampling

**Fig. 1:**
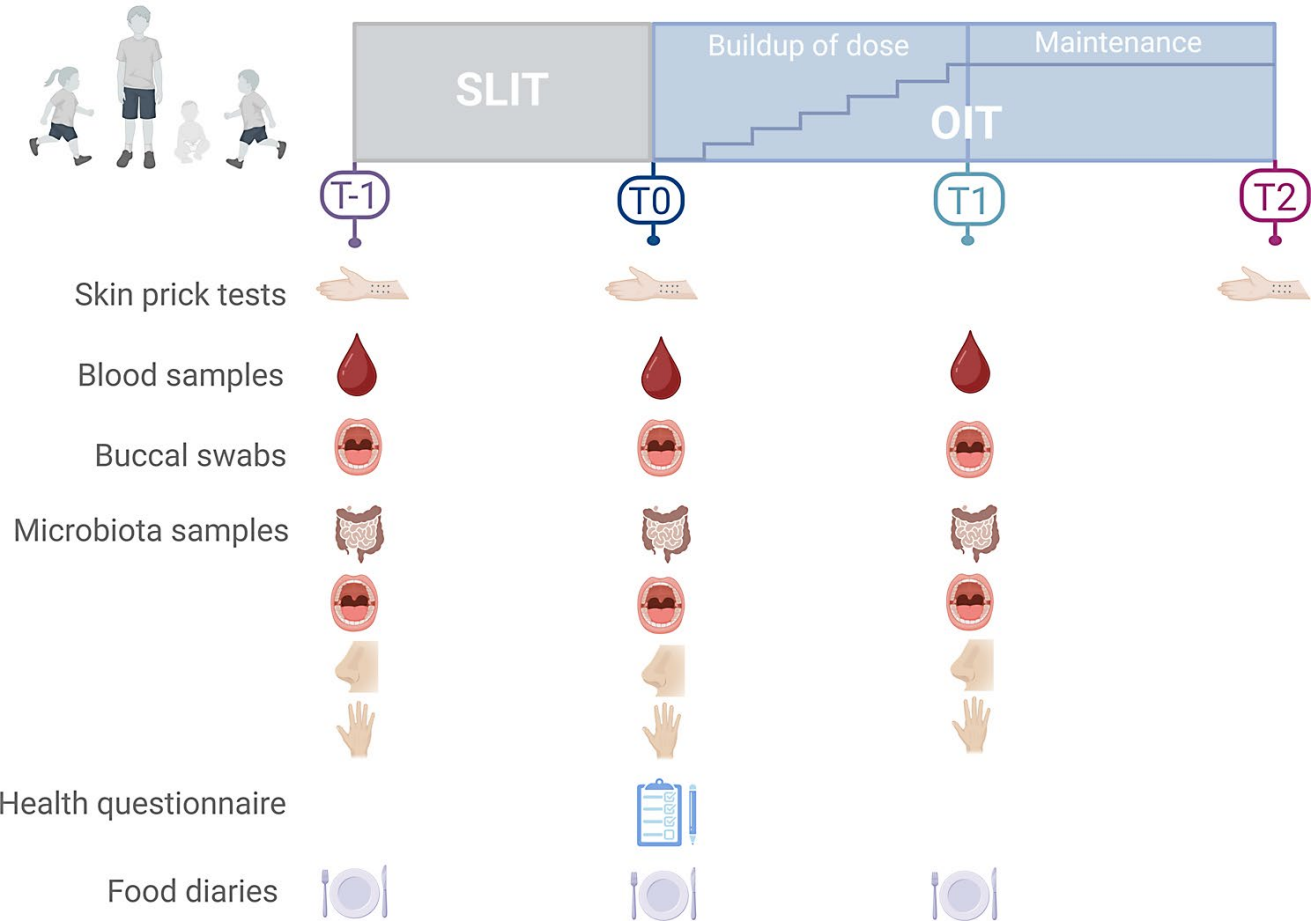
Study protocol of the *Zéro allergie* research clinic. Skin prick tests, food diaries, and various samples are taken before the sublingual immunotherapy (SLIT) (T-1), before the oral immunotherapy (OIT) (T0), and at the end of the buildup of dose (T1)

OIT protocol is depicted in the Fig. 1. Pre-school age participants underwent a preliminary step of SLIT to ensure that their window of opportunity was not missed while waiting for the start of their OIT. Children then underwent OIT dosing protocol with an approximate duration of eight to 18 months followed by a maintenance phase of 18 months. Blood samples, buccal swabs, microbiota samples (buccal, cutaneous, intestinal, and nasal) were collected at the beginning of the SLIT, if applicable, as well as at the start and end of OIT dosing protocol. Blood samples will allow DNA extraction and measurement of DNA methylation and gene expression. Plasma samples will be used to measure metabolite levels. Buccal swabs were carried out to ensure genetic data will be available for all children considering potential difficulties and disincentive associated with blood samples in children. Food diaries were collected at the same time as stool samples to document the children’s diet in relation to gut microbiome. Moreover, a clinical standardized health and environmental questionnaire was administered at the start of OIT. These various samples constitute a biobank for the ZAC and will be used to evaluate the impact of OIT on the immune system, genetics, epigenetics, transcriptomics, metabolomics, and the microbiota. A detailed list of the different samples available before and after OIT is presented in Supplemental Fig. 1.

### Phenotypic characteristics and food allergens sensitization

Phenotypic characteristics of the 183 allergic children and 27 non-allergic siblings in the ZAC are presented in Table 1. A total of 129 children were of preschool age (four years and under), but age ranges from 1 to 16 years. Only a small proportion (5.6%) of children were on omalizumab during OIT. Regarding dosing schedule protocol, a total of 124 children were assigned to the standard dosing protocol with dose increase every two weeks, while 59 children were assigned to the double dosing protocol with dose increase every two months.

**Table 1:** Phenotypic characteristics of children in the Zéro allergie cohort (ZAC)

Characteristics	Allergic children (*n* = 183)	Non-allergic siblings (*n* = 27)
Female sex, n (%)	78 (42.6)	11 (40.7)
Age, mean (range)	4 (1–16)	5 (2–12)
Age, median	3	5
Cesarean delivery, n (%)	49 (26.8)	7 (25.9)
Breastfeeding, n (%)	137^a^ (75.7)	18 (66.7)
Omalizumab, n (%)	10 (5.5)	-
Asthma^b^, n (%)	48^c^ (26.8)	9 (33.3)
Atopic dermatitis^d^, n (%)	155^e^ (85.2)	17 (63.0)
Allergic rhinitis^f^, n (%)	21^a^ (11.6)	4 (14.8)
Pollen allergy^g^, n (%)	26^h^ (14.5)	1^i^ (5.9)
Dosing schedule protocol		
Standard dosing protocol, n (%)	124 (67.8)	-
Double dosing protocol, n (%)	59 (32.2)	-

^a^ Data available for 181 children. ^b^ Diagnosis of asthma by a specialist (paediatrician or pneumologist). ^c^ Data available for 179 children. ^d^ Self-reported history of atopic dermatitis. ^e^ Data avaiblable for 182 children. ^f^ Self-reported history of allergic rhinitis. ^g^ Self-reported history of pollen allergy. ^h^ Data availble for 169 children. ^i^ Data available for 17 children.

**Fig. 2:**
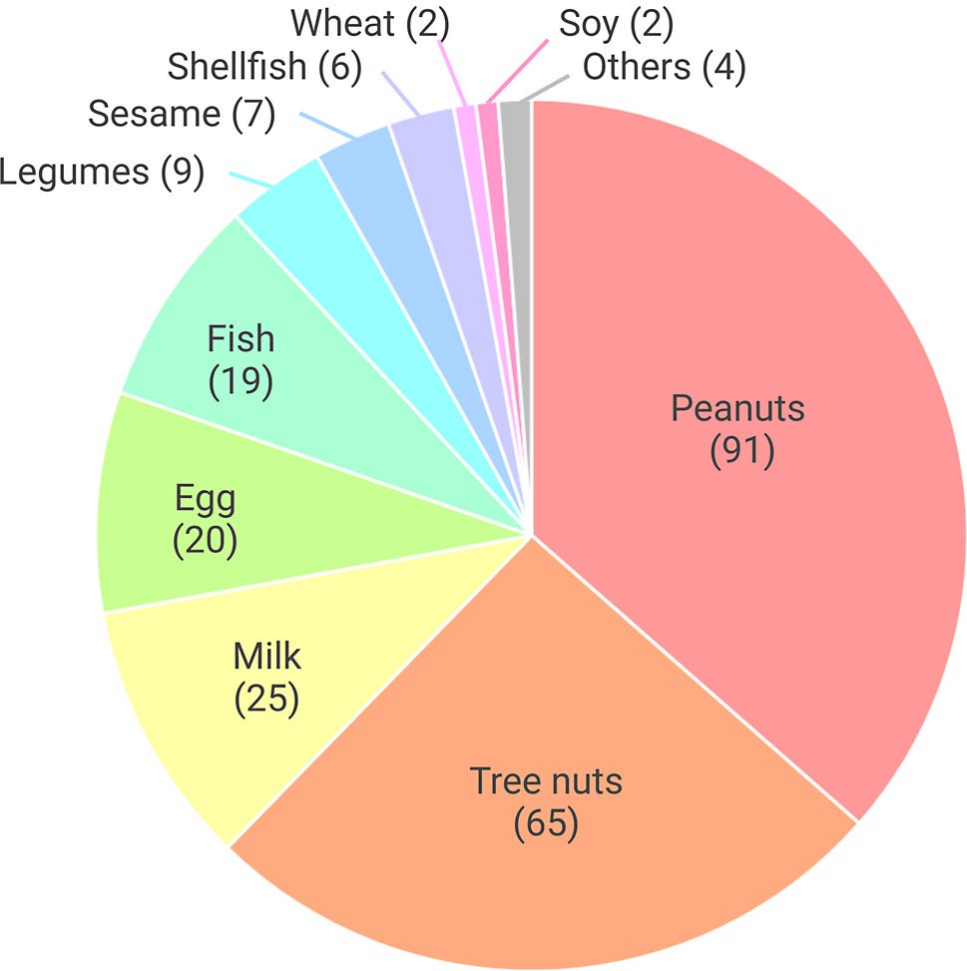
Numbers of food allergens desensitized in oral immunotherapy in children of the *Zéro allergie* cohort

A total of 119 children (65.0%) had one FA, 49 (26.8%) had two FAs, and 15 (8.2%) had three or more. Even if children with more than three FA were to be excluded according to inclusion criteria, some of them developed other FA after OIT started. Food allergens desensitized in the OIT are illustrated in Fig. 2. Peanuts and tree nuts represent most of the food allergens desensitized at the *Zéro allergie* research clinic.

### OIT outcomes

**Fig. 3:**
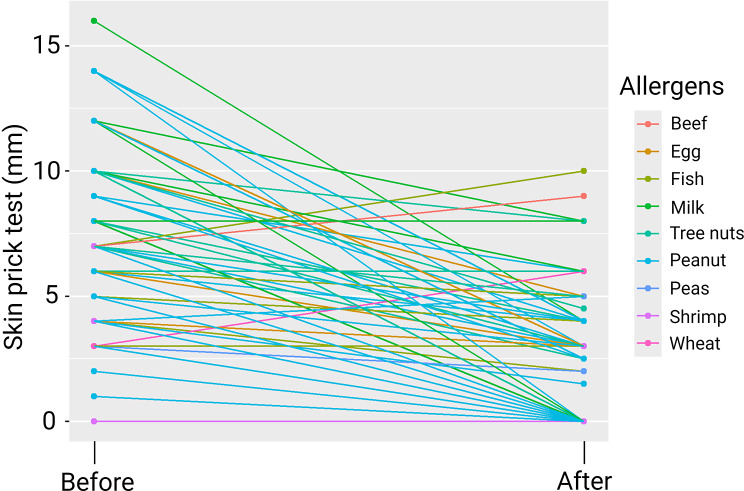
Skin prick tests before and after oral immunotherapy (*n* = 90)

**Fig. 4:**
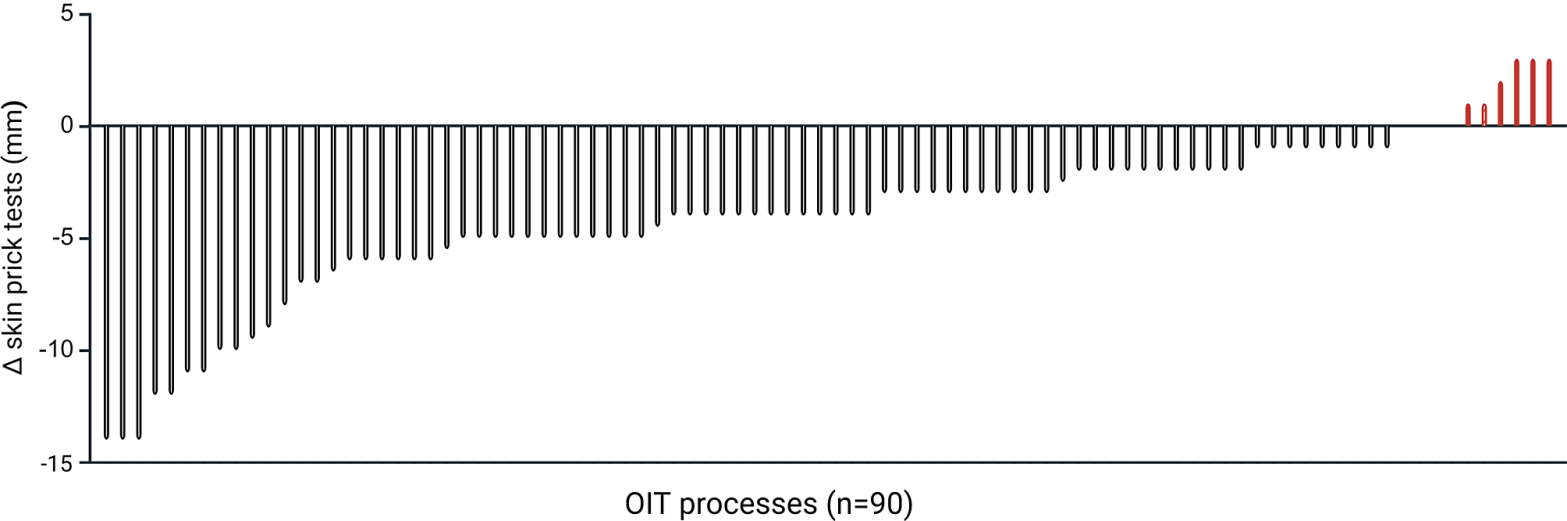
Changes in skin prick tests in 90 oral immunotherapy processes in 67 children since some children followed oral immunotherapy (OIT) for multiple allergens. Bars represent OIT processes

Of the 183 children following OIT, 69 are currently in the OIT dosing protocol and 114 completed it and are in the maintenance phase (clinical desensitization). Children achieving clinical desensitization can consume 400 mg of allergenic food protein, thus enjoying a protection against accidental ingestion of allergen traces. The average duration of OIT was 307.8 ± 116.0 days. A total of 67 out of the 114 children completed the 18-month maintenance phase. The desensitized allergens in these 69 children were beef, egg, fish, milk, tree nuts, peanut, peas, shrimp, and wheat. Some children completed the maintenance phase for more than one allergen, producing results for 90 OIT processes. Skin prick tests before and after the OIT were significantly different (*P* = 0.0048) even when considering effects of sex, age, and type of allergen (*P* = 0.0072). They are illustrated in Fig. 3. Absolute changes in those tests are also depicted as a bar plot in Fig. 4.

Skin prick test wheal diameters decreased for 80 OIT treatments, while they remained stable for four and increased for six. After the 18-month maintenance phase, a total of 41 out of 90 OIT treatments (45.6%) led to complete desensitization where children were able to consume the allergenic food without restriction. For the other 45 OIT treatments (50%), clinical desensitization was also obtained but not complete desensitization. The maintenance phase with daily ingestion of 400 mg of allergen then had to be continued for another 12 months since skin prick tests were still positive.

### Impact on accessibility to OIT

This initiative led to an important reduction of the waiting list for OIT in the SLSJ region. At the inception of the *Zéro allergie* research clinic in January 2020, more than 200 children were waiting an average of two years for OIT. As of June 2024, the clinic’s waiting list included six children with an average waiting time of three months. The clinic will soon be able to alleviate local hospital’s (CIUSSS of SLSJ) waiting list.

## Discussion

FA is a major public health concern with an increasing prevalence and considerable physical health, quality of life, and economic burdens [28]. For Canadian children, the most common physician-reported FA were peanut (0.8% of children), tree nut (0.6%), cow’s milk (0.4%), egg (0.3%), fruit (0.2%), finned fish (0.2%), and shellfish (0.2%) [29]. The distribution of FA in children of the ZAC is consistent with these results. The increasing prevalence of FA may be attributed to a complex interplay of environmental and genetic factors [30]. In children genetically predisposed to allergy, environmental factors such as timing and route of exposure to foods, increased hygiene, and use of antibiotics can increase the risk of FA [30]. Indeed, current guidelines recommend the early introduction of food allergens in the infant diet to prevent FA [31]. On the other hand, the penetration of food allergens through inflamed and disrupted skin barrier of infants with atopic dermatitis leads to food sensitization, the first step in the development of FA [32]. This is part of a pattern of progression of allergic diseases called atopic march, where atopic dermatitis in early childhood progresses with FA, asthma, and allergic rhinitis later in life [33].

The goal of FA management is to empower patients and caregivers to manage the risk of FA reactions as well as improve patients’ sense of control and reduce their anxiety [34]. For many patients, this can be achieved through OIT [35]. However, there are disparities in the access to care, diagnostic, and proper management of FA including OIT [8]. In fact, one of the major problems with OIT is its accessibility, especially outside urban centers [8]. The establishment of the *Zéro allergie* research clinic has greatly increased accessibility to the OIT in the SLSJ region.

So far, a total of 183 children with FA and 27 non-allergic siblings have been recruited in the ZAC. The participation rate was very high (91.2%) for allergic children, which allows us to quickly increase the ZAC sample size. However, it is more difficult to recruit non-allergic siblings since most parents refuse to have samples taken from their non-allergic children. A total of 11 children dropped out of OIT, including two for personal reasons not related to OIT, and nine due to symptoms or difficulties associated with OIT, namely significant allergic symptoms, anxiety, and difficult compliance. These difficulties and challenges are similar to those reported by the international Delphi consensus, which include difficulty with adherence, food aversion, dose-related anxiety, and extended time taking the daily dose [13]. OIT is a long process requiring a significant commitment from both patients and caregivers. This demonstrates the importance of developing new alternative approaches to FA care for individuals who chose not to do OIT or do not have the profile to ensure its success. This also reinforces the need to better understand the underlying mechanisms of OIT and why clinical desensitization is not reached for some children. Considering the dropout rate of 7.3%, OIT allowed to desensitize more than 92% of the children enrolled in the protocol. However, as several children are still in the dosing protocol and recruitment is still ongoing, those percentages are not final for the cohort. Moreover, among the 67 children who completed the 18-month maintenance phase, 88.9% of skin prick tests were reduced. This is in line with the imperfect concordance between skin prick tests results and clinical symptoms of FA [36]. OIT was also associated with the development of complete desensitization in almost half of cases after an 18-month maintenance phase, which is in line with the results of numerous randomized controlled trials on OIT efficacy [10, 37, 38].

In addition to the clinical data acquired during OIT, blood samples, buccal swabs, and microbiota samples (buccal, cutaneous, intestinal, and nasal) are collected at the beginning and at the end of OIT. This will create a unique and rich collection of genetic, epigenetic, transcriptomic, metabolomic, and microbiota data to better characterize children following OIT and understand the underlying mechanisms of FA. It will be possible, for example, to determine whether the genetic profile of children influences the success of OIT. Moreover, DNA methylation changes following OIT will be investigated and linked to gene expression levels. Monitoring plasma metabolites as well as serum total IgE, specific IgE, and IgG levels will allow to follow the desensitization process. To the best of our knowledge, only one study has examined intestinal microbiota changes in children undergoing OIT [21]. The identification of genetic, epigenetic, transcriptomic, metabolomic, and microbial profiles specific to the prognosis of the OIT should make it possible to create predictive tools. This would ultimately contribute to identify children who are most likely to succeed in OIT, which would be highly beneficial considering the long waiting lists. Moreover, it will help identify individuals with a poorer prognosis, in order to discuss risks and benefits so that they can make an informed decision. Ultimately, from a perspective of precision medicine, our study will contribute to the development of new treatment options for children with low chances of success. We are not aware, to the best of our knowledge, of another cohort with similarly extensive biological and clinical data collection, which means that no replication studies are possible for those measures at that time.

The cohort design would allow to study epigenetic, transcriptomic, metabolomic, and microbiota changes not only in OIT but also in SLIT in some children. In addition, the efficacy of both dosing protocols can be evaluated and the response according to omics profiles can be assessed. Finally, this clinical and research initiative has already dramatically reduced the waiting list for OIT in the SLSJ region, allowing more children to access it quickly, thus increasing the chances of treatment success.

## Conclusions

The ZAC recruited, to date, 183 children with FA and 27 non-allergic siblings to study genetic, epigenetic, transcriptomic, metabolomic, and microbial diversity profiles associated with FA and OIT. It constitutes a unique cohort and rich biobank of biological samples, clinical, and phenotypic data to better understand OIT and identify potential biomarkers of success. The *Zéro allergie* research clinic represents an innovative interdisciplinary initiative by researchers, allergists, and paediatricians to make allergy care, including OIT, accessible to a larger portion of the allergic population. Finally, this will also contribute to the development of international research initiatives through international consortiums to address this global public health concern.

## Electronic supplementary material

Below is the link to the electronic supplementary material.

Supplementary Material 1

## Data Availability

The datasets used and/or analysed during the current study are available from the corresponding author on reasonable request.

## Abbreviations

FA: Food allergy
Ig: Immunoglobulin
OIT: Oral immunotherapy
SLIT: Sublingual immunotherapy
SLSJ: *Saguenay–Lac-Saint-Jean*
SU: Sustained unresponsiveness
ZAC: *Zéro allergy* cohort
