# Supplementary material for: Zéro allergie research clinic: a clinical and research initiative in oral immunotherapy for managing IgE-mediated food allergy
Source: Allergy Asthma Clin Immunol. 2024 Nov 2;20:59. doi: 10.1186/s13223-024-00921-8 (PMC11531141; doi:10.1186/s13223-024-00921-8)
Supplement: Supplementary file 1 — Supplementary Material 1 [file 13223_2024_921_MOESM1_ESM.docx]

**Supplementary material**

Tremblay BL et al. *Zéro allergie* research clinic: A clinical and research initiative in oral immunotherapy for managing IgE-mediated food allergy. 2024


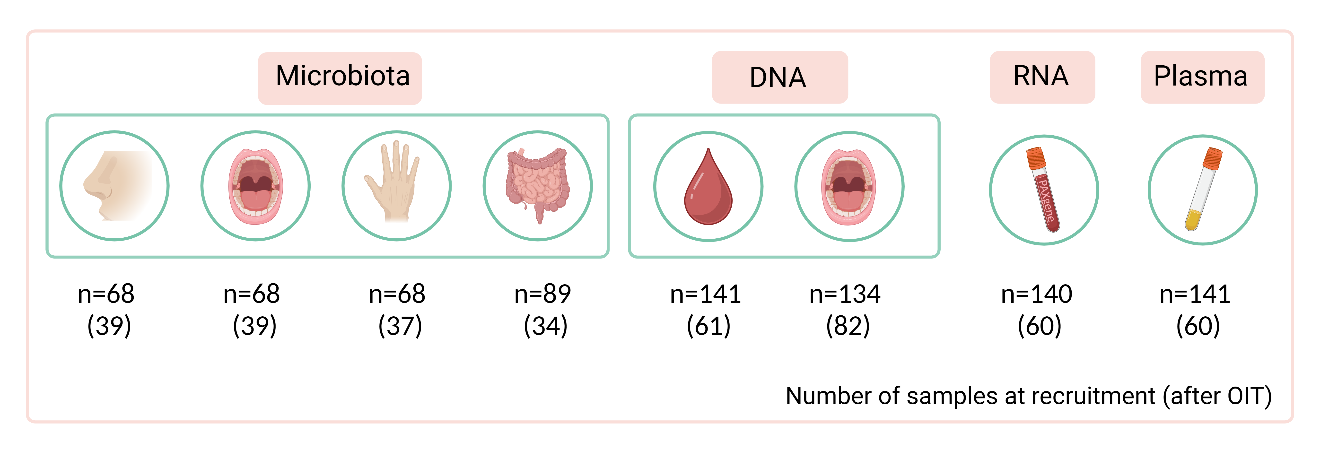


**Supplemental Figure 1**. Samples available from the *Zéro allergie* cohort biobank. Numbers of samples available at recruitment, before oral immunotherapy (OIT) and after OIT (in parentheses) are presented.
